# Effective Thoracic Duct Ligation at T9 for Postoperative Chylothorax: A Case Report

**DOI:** 10.7759/cureus.104582

**Published:** 2026-03-02

**Authors:** Yusuke Nabe, Hiroshi Mizuuchi, Masaaki Inoue, Junichi Yoshida

**Affiliations:** 1 Department of Chest Surgery, Shimonoseki City Hospital, Shimonoseki, JPN

**Keywords:** intrathoracic adhesions, lung cancer surgery, mediastinal lymph node dissection, postoperative chylothorax, thoracic duct anatomy, thoracic duct ligation, video-assisted thoracoscopic surgery

## Abstract

Postoperative chylothorax after lung resection and mediastinal lymph node dissection is a rare complication that may be difficult to manage when conservative treatment is ineffective, and the thoracic duct or leak point cannot be identified intraoperatively. Chylothorax is thought to result from thoracic duct injury associated with mediastinal lymph node dissection. Although surgical thoracic duct ligation is recommended in refractory cases, identification of the leakage site or thoracic duct itself is often challenging, particularly in patients with intrathoracic adhesions or in facilities where lymphangiography cannot be performed. An 82-year-old man underwent thoracoscopic right upper lobectomy with mediastinal lymph node dissection for lung adenocarcinoma with mediastinal invasion after induction chemotherapy. Postoperatively, a pulmonary fistula and chylous effusion developed. Although the chylous effusion initially improved with conservative treatment, including a low-fat diet, massive right pleural effusion recurred after discharge. Pleural fluid examination revealed elevated triglyceride levels, leading to a diagnosis of chylothorax. Conservative treatment with dietary restriction and octreotide was ineffective, and thoracoscopic surgery was performed. During the reoperation, severe intrathoracic adhesions and thickening of the mediastinal pleura were observed due to prior surgery and pleurodesis. The areas of previous mediastinal lymph node dissection were carefully examined; however, no clear lymphatic fistula or thoracic duct was observed. Intraoperative intravenous indocyanine green did not delineate the thoracic duct. Because identification of the thoracic duct at the supradiaphragmatic level was considered difficult, the mediastinal pleura was incised at the T8-T9 level, where the thoracic duct anatomically runs between the azygos vein, descending aorta, and esophagus. The adipose tissue containing the presumed thoracic duct was ligated *en bloc* using silk sutures and resected. Chylous drainage decreased immediately after the procedure, and the chylothorax resolved without recurrence. At the T9 level, posterior mediastinal adipose tissue is relatively limited compared to that at the supradiaphragmatic level, making *en bloc* ligation feasible even when the thoracic duct cannot be directly identified. Ligation of the thoracic duct together with the surrounding adipose tissue at the T9 level may represent an effective alternative surgical option for postoperative chylothorax in cases where lymphangiography is unavailable or where intrathoracic adhesions make conventional thoracic duct ligation difficult.

## Introduction

Chylothorax after lung resection and mediastinal lymph node dissection is reported to occur in 1.4% of patients, with a higher incidence in patients with pathologically diagnosed N2 disease and those undergoing robot-assisted resection [1]. Postoperative chylothorax is thought to result from thoracic duct injury associated with mediastinal lymph node dissection for primary lung cancer, and the most common site of chyle leakage is the #4R lymph node [2]. Chylothorax is more common in the right thoracic cavity [2] but can also occur in the left thoracic cavity [3]. The diagnosis of chylothorax is suggested by milky pleural fluid and supported by pleural triglyceride levels > 110 mg/dL and cholesterol levels below 200 mg/dL [4].

Chylothorax can lead to life-threatening conditions due to severe malnutrition and immunosuppression [1], with the 90-day mortality rate associated with chylothorax after esophagectomy reaching 82% [4]. Chylothorax requires conservative treatment [5], percutaneous thoracic duct embolization [6,7], and surgical treatment [8,9]. At the T9-T10 level, the thoracic duct most consistently courses between the azygos vein and descending aorta, adjacent to the esophagus, whether it is a single, double, or plexiform thoracic duct [10].

The recommended surgical procedure for chylothorax is an open thoracotomy with thoracic duct ligation if the location of the injury is known or mass ligation of the thoracic duct-containing tissue above the diaphragm or below the aortic arch [11]. Video-assisted thoracoscopic ligation is widely used when feasible. Several reports have indicated that initial thoracic duct ligation above the right diaphragm is ineffective because of the presence of a double thoracic duct or a few hidden branches [3]. Mass ligation of the tissue containing the thoracic duct directly above the diaphragm is an alternative surgical method; however, several reports have suggested that permanent occlusion may not be achieved because the thoracic duct is not individually isolated [9].

Lymphangiography [12] and intraoperative staining have been used to identify leakage sites [13-15]. However, only a limited number of facilities can perform lymphangiography or intraoperative staining. Alternative strategies are needed when supradiaphragmatic ligation or image-assisted approaches are not possible. En bloc ligation at the T9 level, taking advantage of anatomical characteristics, may be effective. Here, we report a case of postoperative chylothorax in which the thoracic duct could not be identified intraoperatively; however, the condition was resolved by ligating the thoracic duct en bloc with fatty tissue at the T9 level.

## Case presentation

An 82-year-old male patient with a history of smoking three cigarettes daily (~9 pack-years) from the age of 20 to 81 years presented with a year-long cough. A chest radiograph performed for medical examination revealed an abnormal shadow in the right upper lung field. Computed tomography (CT) revealed suspected right upper lobe lung cancer, and the patient was referred to our department.

**Figure 1:**
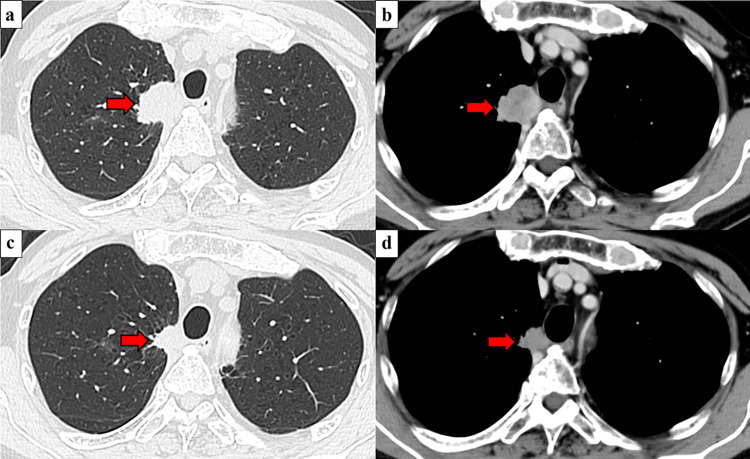
Preoperative CT findings. a. Before chemotherapy: The tumor size was 28 × 26 mm; no obvious lymph node metastases were observed. b. Before chemotherapy: Mediastinal infiltration was also observed. c. After chemotherapy: The tumor had shrunk to 18 × 17 mm. d. After chemotherapy: Mediastinal infiltration remained. The red arrow in each panel indicates the tumor.

Contrast-enhanced chest CT revealed a 28 × 26 mm nodule in the right upper lobe of the lung with infiltration into the mediastinum (Figure 1). Bronchoscopy was performed, and a cytological diagnosis of adenocarcinoma was made. FDG-PET/CT and brain magnetic resonance imaging (MRI) revealed a diagnosis of cT4N0M0, stage IIIA. As the diagnosis was based on cytology and the tumor volume was relatively small, PD-L1 and genetic testing could not be performed. Preoperative treatment consisted of cisplatin (75 mg/m2, 90 mg/body, 75% dose), pemetrexed (500 mg/m2, 630 mg/body, 80% dose), and pembrolizumab (200 mg/body). After the first cycle, a persistent Grade 3 rash developed, and chemotherapy was discontinued from the second cycle onward. The best overall response was a partial response (PR); however, mediastinal infiltration persisted, resulting in a post-treatment disease stage of ycT4N0M0, stage IIIA. Tumor downstaging was not possible. Because a histological diagnosis had not yet been made, surgery was selected for both diagnosis and treatment. Thoracoscopic right upper lobectomy and systematic mediastinal lymph node dissection (ND2a-1; stations #2R and #4R) were performed. The tumor was firmly attached to the superior border of the azygos vein and showed mediastinal infiltration. However, complete resection was possible without concomitant azygos vein resection. Mediastinal lymph node dissection was performed using Harmonic® ACE (HA) (Ethicon Endo-Surgery, USA) with #2R and #4R dissections.

**Figure 2:**
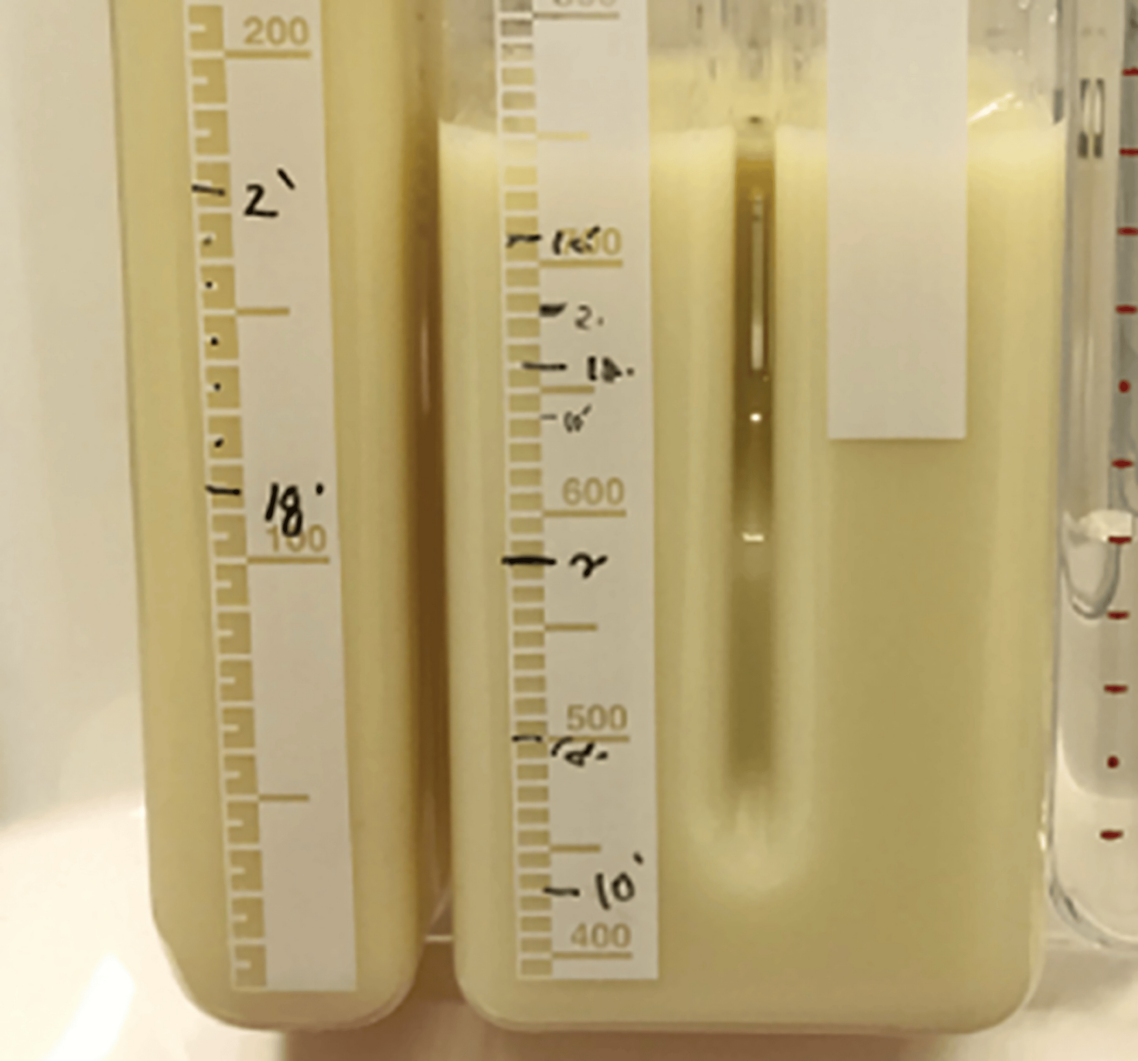
Pleural effusion findings. After right thoracic drainage, a milky white pleural effusion was confirmed.

Postoperatively, a pulmonary fistula and chylous pleural effusion were observed. The maximum amount of pleural effusion was 70 ml/d. Pleurodesis (minocycline 200 mg) was performed on postoperative day (POD) 4 to treat the pulmonary fistula; however, the pulmonary fistula persisted, and thoracoscopic pulmonary fistula repair was performed on POD 9. No clear lymphatic fistula was identified at the time of pulmonary fistula repair, and the chylous effusion improved with conservative treatment and a low-fat diet. Following repair, the patient was discharged on POD 25. However, on POD 43, the patient developed fatigue and returned to our department. CT revealed massive right pleural effusion, and the patient was admitted immediately after right-sided pleural drainage. The pleural effusion reached a maximum of 520 ml/d. The pleural effusion was chylous (Figure 2), and pleural fluid examination revealed an elevated triglyceride level of 263 mg/dL, leading to a diagnosis of chylothorax.

The results of the pleural fluid examination are shown in Table 1. No universally established reference ranges exist for pleural fluid parameters. Pleural fluid values were interpreted using established diagnostic criteria rather than serum reference ranges [4,16-18]. Pleural fluid cytology results were negative.

**Table 1:** Pleural fluid analysis. LD, lactate dehydrogenase; Alb, albumin; AMY, amylase; Glu, glucose; TG, triglyceride; TP, total protein; PCR, polymerase chain reaction; ADA, adenosine deaminase. No universally established reference ranges exist for pleural fluid parameters. Pleural fluid values were interpreted using established diagnostic criteria rather than serum reference ranges [4,17-19]. * The Light's criteria are used to classify pleural effusions [18]. To be considered an exudate, at least one of the following conditions must be met: 1) pleural fluid protein/serum fluid protein ratio > 0.5, 2) pleural fluid lactate dehydrogenase (LDH)/serum fluid LDH ratio > 0.6, or 3) pleural fluid LDH > 2/3 of the upper limit of normal serum LDH [18].

Parameter	Result	Reference range	Unit
pH	7.2	≧7.2 [18]	—
Rivalta reaction	Negative	—	—
Lactate dehydrogenase (LDH)	186	— *	U/L
Albumin (Alb)	1.7	≦1.2 [17]	g/dL
Amylase (AMY)	49	≦ 110 [18]	U/L
Glucose (Glu)	110	≧60 [19]	mg/dL
Triglyceride (TG)	263	≦ 110 [4]	mg/dL
Total protein (TP)	3.0	— *	g/dL
Mycobacterium tuberculosis PCR	Negative	—	—
Adenosine deaminase (ADA)	16.1	≦40 [18]	U/L

Conservative treatment with low-fat food and octreotide was attempted, but no improvement was observed. Thoracoscopic thoracic duct ligation was performed on POD 58. Milk was administered via a gastric tube immediately before surgery. The procedure was performed under general anesthesia, with the patient in the left lateral position. Because of prior surgeries and pleurodesis, adhesions were observed within the thoracic cavity, and the mediastinal pleura was thickened. Areas where the lymph nodes had been dissected (#2R and #4R) during the previous surgery, and the surrounding mediastinum were observed. However, it was difficult to identify the site of lymphatic leakage, and an intraoperative intravenous injection of indocyanine green (ICG) failed to detect the thoracic duct.

**Figure 3:**
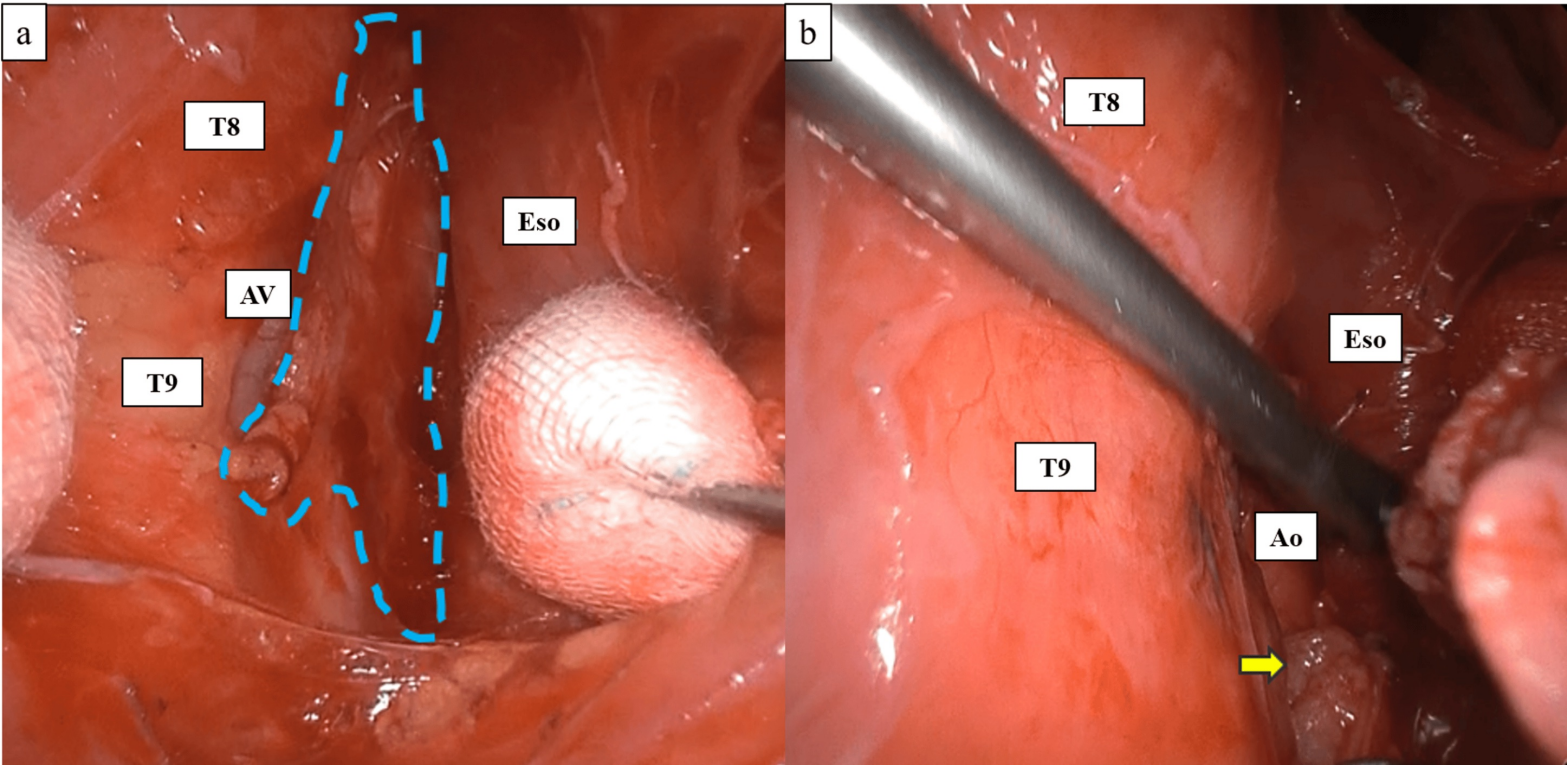
Intraoperative findings. a. Fat tissue containing the thoracic duct before resection. The area surrounded by the dotted line represents the fat tissue through which the thoracic duct runs. b. Findings after cutting the adipose tissue, including the thoracic duct. The adipose tissue, including the thoracic duct, was double-ligated with a 2-0 silk thread at the T9 level, and the area between them was cut with a harmonic. The descending aorta was confirmed after cutting. Ao, aorta; AV, azygos vein; Eso, esophagus; T8, 8th thoracic vertebra; T9, 9th thoracic vertebra. The yellow arrow indicates the adipose tissue stump with the thoracic duct.

Adhesions made it challenging to identify the thoracic duct at the supradiaphragmatic level. The mediastinal pleura behind the hilum was incised at the T8/T9 level, and the tissue bundle within the azygos-aorta-esophagus triangle was doubly ligated using silk sutures proximally and distally and then divided at the T9 level. The segment between the ligatures was then resected using a Harmonic® ACE (HA) (Ethicon Endo-Surgery, USA) (Figure 3), resulting in a reduction in the chylous exudate.

**Figure 4:**
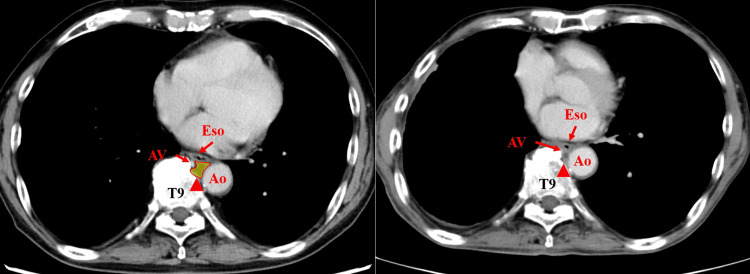
Comparison of CT findings before and after thoracic duct ligation. a. T9 levels (before lung cancer surgery): The red triangular arrow shows the adipose tissue containing the thoracic duct surrounded by the azygos vein, descending aorta, and esophagus. b. T9 level (after thoracic duct ligation): The red triangular arrow shows the postoperatively observed defect in the adipose tissue surrounded by the azygos vein, descending aorta, and esophagus. Ao, aorta; AV, azygos vein; Eso, esophagus; T, thoracic vertebra.

The right chest tube was removed on POD 7 following thoracic duct ligation. On POD 9, an increased inflammatory response was observed, leading to suspicion of pneumonia and empyema, and ampicillin/sulbactam was initiated, which was discontinued on POD 17. After rehabilitation, the patient was discharged on POD 30. CT scans obtained before lung cancer surgery and three months after thoracic duct ligation are shown in Figure 4. Following thoracic duct ligation, an area corresponding to the presumed resected fatty tissue was absent.

## Discussion

Malignant tumors are known to induce lymphangiogenesis and promote lymph node metastasis through the release of growth factors such as VEGF-C [19]. In our case, the tumor invaded the mediastinum, potentially associated with increased peritumoral lymphatic channels. Octreotide reduces splanchnic blood flow and intestinal fat absorption, thereby decreasing lymph flow. Chylothorax after lung resection can be treated conservatively in 90% of cases by combining octreotide administration with adherence to an MCT diet [1]. However, in this case, treatment with a combination of octreotide and a low-fat diet was unsuccessful.

Although previous reports indicate that preoperative lymphangiography can be effective [12], the procedure is not available at our institution owing to the absence of experienced specialists.

We suspected that the leakage was due to damage to the lymphatic vessels during mediastinal lymph node dissection. However, despite observing the area around the mediastinal lymph node dissection and within the mediastinum, we were unable to identify the site of the leakage. In previous cases, the thoracic duct was identified using indocyanine green (ICG), which was consistently administered to the inguinal lymph nodes [13-15]. Intranodal (inguinal) ICG injection was not available/feasible at our institution; therefore, IV ICG was attempted but did not provide lymphatic-specific contrast. In this case, ICG was administered intravenously during surgery; however, it stained not only the lymphatic vessels but also the veins in the thoracic cavity, making it difficult to identify the thoracic duct.

Although multiple case reports have documented the effectiveness of thoracic duct ligation in treating chylothorax, all of these cases involved ligation or clipping of the duct above the diaphragm [3,9]. However, some reports have indicated that supradiaphragmatic duct ligation does not always yield satisfactory results [3]. The cisterna chyli is not present in all patients, and in those without it, the thoracic duct is formed by the confluence of a less distinct lymphatic plexus [20]. As mediastinal fat tissue is abundant at the supradiaphragmatic level, en bloc ligation with the surrounding fat is difficult unless the thoracic duct is clearly identified. Furthermore, in cases with intrathoracic adhesions, such as the present case, it is difficult to identify the thoracic duct at the supradiaphragmatic level.

As previously mentioned, the thoracic duct is always located between the left anterolateral border of the azygos vein and the right border of the aorta between the T9 and T10 levels, regardless of whether it is a simple, double, or plexiform thoracic duct [10]. At the T9 level, as shown in the CT images in this case, the space bounded by the azygos vein, descending aorta, and esophagus was limited, allowing anatomical identification of the fatty tissue containing the thoracic duct. In contrast to the supradiaphragmatic level, the reduced volume of mediastinal fat at this level enables en bloc ligation of the mediastinal fat tissue and the thoracic duct. This method does not require specialized procedures such as lymphangiography or the use of drugs such as ICG and is therefore likely to be feasible in a greater number of institutions.

The approach of en bloc ligation of the thoracic duct with surrounding fatty tissue at the T9 level may represent a useful alternative for facilities unable to perform lymphangiography or in cases where intrathoracic adhesions make identification of the thoracic duct at the supradiaphragmatic level difficult.

A limitation of this report is that its conclusions are based on a single case from our center. Further case accumulation and multicenter prospective studies are needed to validate the effectiveness of this surgical approach.

## Conclusions

Patients with lung cancer with mediastinal invasion are considered to be at an increased risk of postoperative chylothorax owing to lymphatic proliferation. However, facilities capable of performing lymphangiography are limited, and the thoracic duct may not always be identified intraoperatively. At the T9 level, where there is less adipose tissue than at the supradiaphragmatic level, en bloc ligation of the thoracic duct together with the surrounding adipose tissue along its presumed anatomical course may be effective.
